# A new species of the genus *Henricia* (Asteroidea, Spinulosida, Echinasteridae) from South Korea

**DOI:** 10.3897/zookeys.997.52703

**Published:** 2020-11-25

**Authors:** Michael Dadole Ubagan, Taekjun Lee, Philjae Kim, Sook Shin

**Affiliations:** 1 Department of Animal Biotechnology & Resource, Sahmyook University, Seoul 01795, South Korea; 2 Marine Biological Resource Institute, Sahmyook University, Seoul 01795, South Korea; 3 Division of Ecological Conservation, Bureau of Ecological Research, National Institute of Ecology, Seocheon-gun, Choongnam 33657, South Korea

**Keywords:** Distribution, DNA barcoding, *Henricia epiphysialis***sp. nov.**, morphology, taxonomy

## Abstract

A new species of the genus *Henricia* Gray, 1840 that belongs to the family Echinasteridae is described from South Korea. *Henricia epiphysialis***sp. nov.** has epiphyseal ossicles at the ends of the abactinal and lateral plates, and the abactinal and lateral spines form a hooked crown. The partial sequence of the mitochondrial *COXI* gene (537 bp) of *H. epiphysialis***sp. nov.** was obtained, and the new species was morphologically and genetically compared with other related *Henricia* species.

## Introduction

Echinasteridae Verrill, 1867 is the only family that belongs to the order Spinulosida Perrier, 1884. This family comprises eight accepted genera: *Aleutihenricia* Clark & Jewett, 2010; *Dictyaster* Wood-Mason & Alcock, 1891; *Echinaster* Müller & Troschel, 1840; *Henricia* Gray, 1840; *Metrodira* Gray, 1840; *Odontohenricia* Rowe & Albertson, 1988; *Plectaster* Sladen, 1889; and *Rhopiella* Fisher, 1940 (Mah 2019). Most of the species are found in genera *Echinaster* and *Henricia*.

A total of 11 species that belong to *Aleutihenricia* or *Henricia* have been reported in South Korea: *Aleutihenricia beringiana* Djakonov, 1950 and 10 *Henricia* species, namely, *Henricia anomala* Hayashi, 1973; *Henricia elachys* Clark & Jewett, 2010; *Henricia leviuscula* Stimpson, 1857; *Henricia nipponica* Uchida, 1928; *Henricia ohshimai* Hayashi, 1935; *Henricia pachyderma* Hayashi, 1940; *Henricia pacifica* Hayashi, 1940; *Henricia regularis* Hayashi, 1940; *Henricia reniossa* Hayashi, 1940; and *Henricia sanguinolenta* O.F. Müller, 1776 (Shin 2010; Shin and Ubagan 2015a, b; Ubagan and Shin 2016, 2019a, b, c; Taekjun and Shin 2020). Most species recorded in South Korea including *H. leviuscula*, *H. nipponica*, *H. pachyderma*, *H. regularis*, *H. reniossa*, and *H. sanguinolenta*, are distributed in the East Sea. *Henricia* species can be distinguished by the ratio of arm to disk, shape and number of abactinal spines, and shape of the skeletal plates.

In DNA barcoding, sequence variation in a 658 bp region of the mitochondrial cytochrome *c* oxidase subunit I (*COXI*) gene is used for specimen identification and species discovery (Hebert et al. 2003). An integrative approach to taxonomy (i.e., using morphological characteristics from preserved specimens as well as one to several genes) has become necessary for assessing species diversity and species boundaries (Puillandre et al. 2012).

In this study, we identified a new species that belongs to the genus *Henricia* collected from waters adjacent to the East Sea, South Korea, and performed detailed morphological and molecular mitochondrial sequence analyses. This paper aims to extend the taxonomical insights to *Henricia* species in South Korea by providing a complete description of this new species.

## Materials and methods

**Figure 1.**
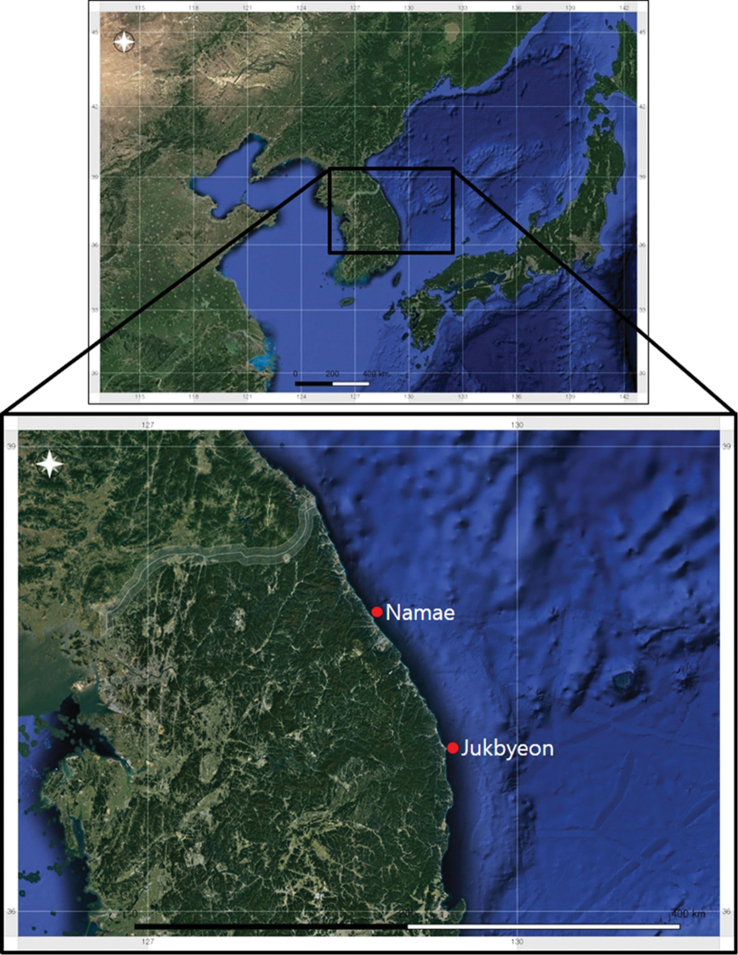
Map of Korea showing the collection sites of *Henricia epiphysialis* sp. nov. in the East Sea, South Korea.

In May and December 2014, sea stars were collected from the East Sea in South Korea by using fishing nets (Fig. 1). The collected specimens were preserved in 95% ethanol and deposited at the National Institute of Biological Resources (NIBR) and Marine Echinoderm Resource Bank of Korea (MERBK), South Korea. The external features of the specimens were observed using a stereomicroscope, and the specimens were identified on the basis of morphological characteristics such as the size of the disk, R/r ratio (R: length of arm; r: radius of the disk), size of the upper and proximal portions of arms, number of abactinal spines, shape of abactinal and actinal skeletons, and number of adambulacral spines. For observing the detailed structures of the specimens such as the shape of the spines and skeletal plates, sodium hypochlorite (5.25% solution) was applied carefully to dissolve the skin (Shin 2010). Then, the specimens were washed with water and observed using the stereomicroscope. The important morphological characteristics of the specimens were photographed using a scanning electron microscope (JEOL JSM-6510), stereomicroscope (Nikon SMZ1000), and digital camera (Nikon D7000). Abbreviations for the measurements were those used by Shin and Ubagan (2015a, b).

Total genomic DNA was isolated from ethanol-preserved tube feet tissue by using a DNeasy blood and tissue DNA isolation kit (Qiagen), according to the manufacturer instructions. The genomic DNA quality and concentration were determined using a Nanodrop ND-1000 spectrophotometer (Thermo Fisher Scientific). All genomic DNA samples were stored at −20 °C until further use. The partial sequence of the mitochondrial *COI* gene (658 bp) was amplified using a pair of primers, LCOech1aF1 (Layton et al. 2016) and HCO2198 (Folmer et al. 1994). PCR was performed using a 25 µL reaction mixture containing 2.5 µL of 10× Ex Taq Buffer containing 20 mM MgCl_2_ (Clontech), 1 µL of 2.5 mM dNTPs (Clontech), 1 µL of each primer at 10pmol, 1.5 µL of the template DNA, 0.3 µL of 5 U/µL Taq polymerase (Clontech), and 17.7 µL of distilled water. The PCR conditions were as follows: initial denaturation at 95 °C/3 min, followed by 35 cycles of denaturation at 95 °C/30 s, annealing at 52 °C/90 s, extension at 72 °C/90 s, and a final extension at 72 °C/7 min. The PCR product quality was determined using electrophoresis with a 1.5% agarose gel stained with ethidium bromide. The PCR products were directly sequenced in both directions by using ABI Big Dye Terminator kits (Applied Biosystems) and ABI 3730XL DNA Analyzer. To construct a neighbor-joining (NJ) tree, *COX1* sequences (189 and 537 bp) from the *Henricia* species dataset, including one sequence of the new species (GenBank accession No. MT086587), were used (Table 1). Four species of genus *Echinaster* were used as outgroups (Table 1). Pairwise distances were calculated using MEGA 7.0 and the Kimura-2-parameter model (Kumar et al. 2016). The gaps and missing data were removed, and the bootstrap analysis was performed with 1000 replicates.

**Table 1.** List of *Henricia* species and GenBank accession numbers of *COX1* gene used in this study.

Species	GenBank No.	Dataset		References
		189 bp	537 bp	
*H. compacta* (Sladen, 1889)	KT268147	+		Lopes et al. 2016
*H. epiphysialis* sp. nov.	MT086587	+	+	present study
*H. hayashii* Djakonov, 1961	LC336732	+	+	Wakita et al. 2019
*H. hedingi* Madsen, 1987	KY853274	+		Knott et al. 2018
*H. kinkasana* Hayashi, 1940	LC336731	+	+	Wakita et al. 2019
*H. leviuscula* (Stimpson, 1857)	MK947912	+	+	Lee and Shin 2019
*H. lisa* Clark, 1949	KY853275	+	+	Knott et al. 2018
*H. nipponica* Uchida, 1928	LC336733	+	+	Wakita et al. 2019
*H. obesa* (Sladen, 1889)	KT268148	+		Lopes et al. 2016
*H. oculata* (Pennant, 1777)	KT268151	+	+	Knott et al. 2018
*H. ohshimai* Hayashi, 1935	LC336735	+	+	Wakita et al. 2019
*H. ohshimai* Hayashi, 1935	LC336736	+	+	Wakita et al. 2019
*H. pachyderma* Hayashi, 1940	MT079801	+	+	Lee and Shin 2020
*H. perforata* (Müller, 1776)	KY853302	+	+	Knott et al. 2018
*H. pertusa* (Müller, 1776)	KY853286	+	+	Knott et al. 2018
*H. regularis* Hayashi, 1940	LC336739	+	+	Wakita et al. 2019
*H. reniossa* Hayashi, 1940	LC336740	+	+	Wakita et al. 2019
*H. reticulata* Hayashi, 1940	LC336737	+	+	Wakita et al. 2019
*H. sanguinolenta* (Müller, 1776)	HM542200	+		Lopes et al. 2016
*H. sanguinolenta* (Müller, 1776)	KY853253	+	+	Knott et al. 2018
*H. spongiosa* (Fabricius, 1780)	KY853268	+	+	Knott et al. 2018
*H. tumida* Verrill, 1909	LC336747	+	+	Wakita et al. 2019
*Henricia* sp. 1	LC336744	+	+	Wakita et al. 2019
*Henricia* sp. 2	LC336742	+	+	Wakita et al. 2019
*Henricia* sp. 3	LC336743	+	+	Wakita et al. 2019
*Henricia* sp. 4	LC336741	+	+	Wakita et al. 2019
*Henricia* sp. 5	LC336738	+	+	Wakita et al. 2019
*Henricia* sp. 6	LC336745	+	+	Wakita et al. 2019
*Henricia* sp. 7	LC336746	+	+	Wakita et al. 2019
*Henricia* sp. 8	LC336730	+	+	Wakita et al. 2019
*Henricia* sp. 9	KY853310	+	+	Knott et al. 2018
*Echinaster brasiliensis* Müller & Troschel, 1842	MG636999	+	+	Seixas et al. 2018
*E. callosus* Marenzeller, 1895	KT268121	+		Lopes et al. 2016
*E. luzonicus* (Gray, 1840)	KT268137	+		Lopes et al. 2016
*E. sepositus* (Retzius, 1783)	LC336729	+	+	Wakita et al. 2019

## Taxonomic results

### Phylum Echinodermata Bruguière, 1791

**Class Asteroidea de Blainville, 1830**

**Order Spinulosida Perrier, 1884**

**Family Echinasteridae Verrill, 1867**

**Genus *Henricia* Gray, 1840**

#### Henricia epiphysialis sp. nov.

Taxon classificationAnimaliaSpinulosidaEchinasteridae

E0D8BF96-D976-5387-B119-7D70CA5D7C5B

http://zoobank.org/E50768C6-B625-43ED-82E1-6D581E38975B

Figs 2 , 3 , 4

##### Material examined.

***Holotype***: South Korea • 1 specimen; waters adjacent to Namae, 37°55'57.31"N, 128°48'45.58"E; 40 m; 28 May 2014; S. Shin and T. Lee; fishing net; MERBK–A–1255. ***Paratypes***: South Korea • 1 specimen; waters adjacent to Jukbyeon, 37°3'32.49"N, 129°26'14.57"E; 100 m; 19 Dec. 2014; S. Shin and T. Lee; fishing net; NIBRIV0000837785. 1 specimen; waters adjacent to Namae, 37°55'57.31"N, 128°48'45.58"E; 40 m; 28 May 2014; S. Shin and T. Lee; fishing net; MERBK–A–1256.

##### Diagnosis.

Regular size, R/r = 4.9–5.4, abactinal plates crowded with 11–40 spines, abactinal and lateral spines forming distinct hooked crown, epiphyseal ossicles formed at ends of abactinal and lateral plates, one to three papulae, marginal and ventrolateral series distinguishable, adambulacral plates bearing 10–14 slender spines.

##### Description.

**Figure 2.**
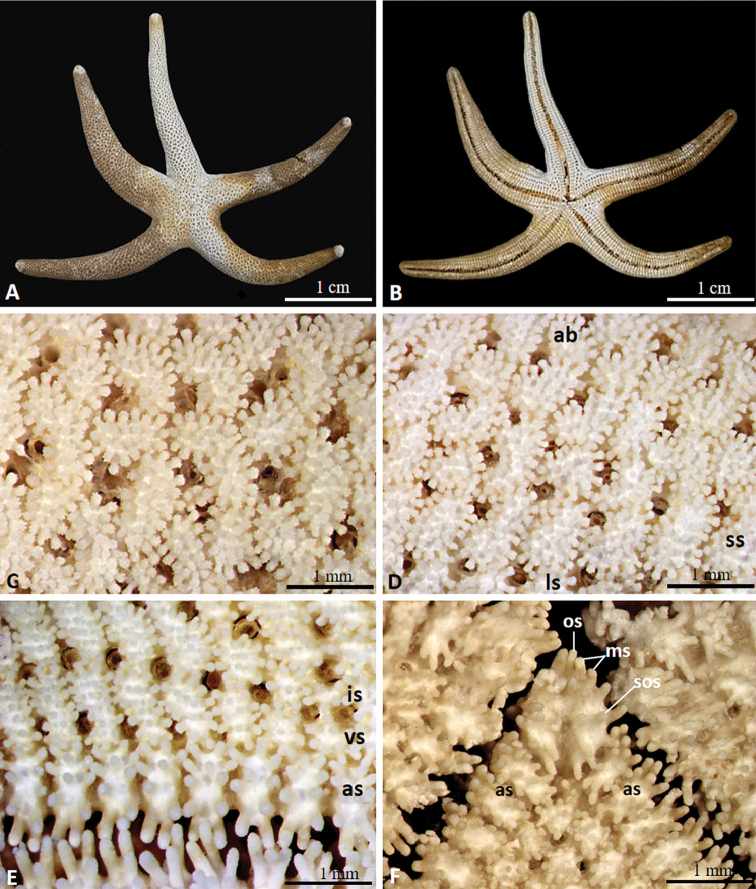
External characteristics of *Henricia epiphysialis* sp. nov. **A** abactinal side **B** actinal side **C** abactinal spines **D** spines on lateral side of arm **E** adambulacral spines **F** oral part. Abbreviations: **ab** abactinal side **ls** lateral side **ss** superomarginal spines **is** inferomarginal spines **vs** ventrolateral spines **as** adambulacral spines **os** oral spines **ms** marginal spines **sos** sub-oral spines.

**Figure 3.**
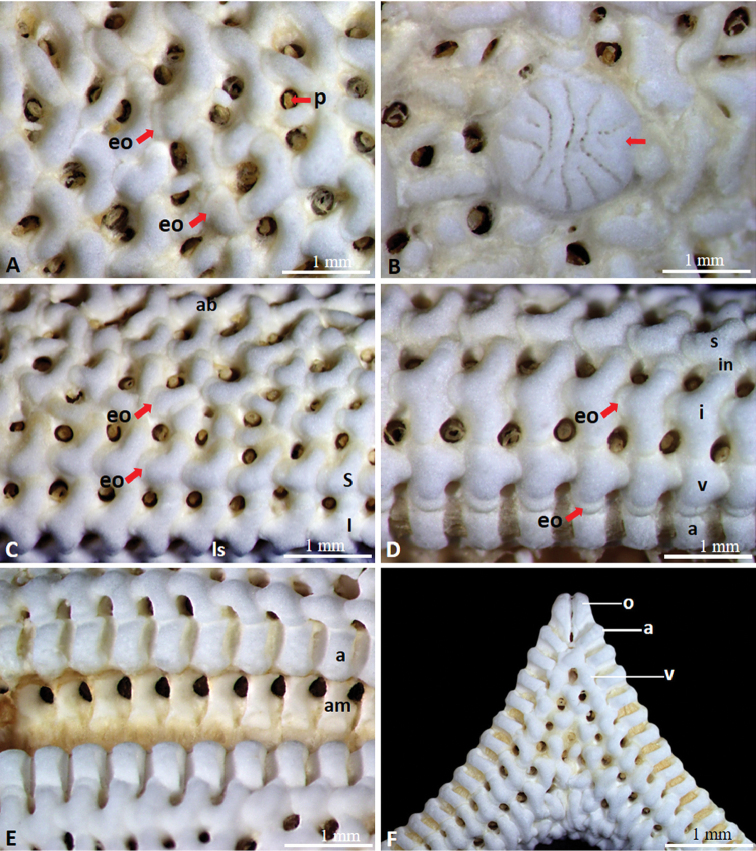
Denuded skeleton of *Henricia epiphysialis* sp. nov. **A** abactinal plates **B** madreporite (arrow) **C** part of abactinal and lateral side of arm **D** plates on the lateral and actinal side of arm **E** actinal plates **F** oral part. Abbreviations: **ab** abactinal side **ls** lateral side **eo** epiphyseal ossicles **p** papula **s** superomarginal plates **in** intermarginal plates **i** inferomarginal plates **v** ventrolateral plates **a** adambulacral plates **am** ambulacral plates **o** oral plates.

**Figure 4.**
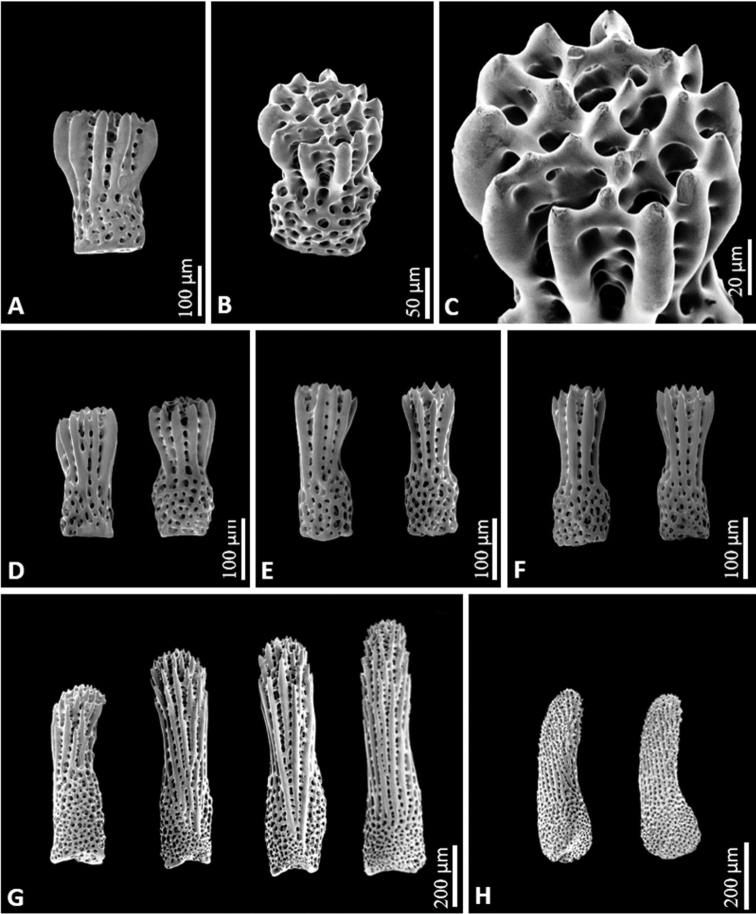
SEM images of the spines of *Henricia epiphysialis* sp. nov. **A, B, C** abactinal spines **D** superomarginal spines **E** inferomarginal spines **F** ventrolateral spines **G** adambulacral spines **H** furrow spines.

***Holotype*.** (Figs 2–4) Size. R = 51 mm, r = 10 mm, R/r = 5.1.

Arms five, semi-cylindrical, gradually tapering to tips (Fig. 2A–B). Abactinal paxillae formed in group with evenly spaced spinulation, bearing 11–40 spines with serrated tips (Fig. 2C). Denuded abactinal spines forming hooked crown composed of nine to 11 large hook-shaped spinules enclosing nine to 12 small connected apical tips (Fig. 4A–C). Paxillae on lateral side of arms similar to abactinal paxillae (Fig. 2D). Denuded abactinal plates reniform, usually connected to end of other plate in mid convex part of plate, larger than papular areas, partially enclosing papular area on concave side of plate. Papular areas narrow, containing one to three papulae in an area. Some papular areas divided by small ossicles (Fig. 3A). Almost every skeletal plate aside from adambulacral plates was observed bearing epiphyseal ossicles at ends of plate (Fig. 3A, C, D). Madreporite circular in form, slightly elevated, bearing spines same as adjacent spines (Fig. 3B). Shape of spines on lateral side nearly similar to that of abactinal spines (Fig. 4D–F). Superomarginal, inferomarginal, and ventrolateral plates well defined forming elongated cross shape and arranged in rows showing consistent series (Fig. 3D). Superomarginal plates bearing 12–28 spines, bend upward toward base of arm in crescentic form, and reach tip of arm (Figs 3C–D, 4D). Intermarginal plates forming small elongated shape, extending near half of arm (Fig. 3D). Inferomarginal plates longer than superomarginal and ventrolateral plates, bearing 34–45 spines, reaching tip of arm (Figs 3C–D, 4E). Ventrolateral plates forming a rounded cross shape, bearing 21–25 spines, reaching near tip of arm, epiphyseal ossicles forming a knob-like connection to adambulacral plates, extending to middle part of arm (Figs 3D, 4F). Adambulacral plates forming semi-rounded shape, bearing 10–14 slender, thorny spines, arranged in two transverse series (Figs 3D–E, 4G), articulated with ambulacral plates (Fig. 3E). Furrow spine single, somewhat curved (Fig. 4H). Oral part bearing two slender, bluntly pointed oral spines, with six or seven marginal spines, and five or six sub-oral spines similar to adambulacral spines (Fig. 2F). Paired oral plates forming a slightly elongated triangular shape, articulated with first pair of adambulacral plates. Plates of inter-radial area slightly compact (Fig. 3F).

***Paratypes*.** Size. R = 39 mm, r = 8 mm, R/r = 4.9; R = 60 mm, r = 11 mm, R/r = 5.4.

##### Etymology.

The specific name is derived from the Latin “epiphysialis,” which means the end part of a long bone.

##### Ecology.

This species is found on hard substrates (rocky bottom) from a shallow water of a depth of 40 m to 100 m.

##### Distribution.

South Korea (East Sea).

### Molecular results

**Figure 5.**
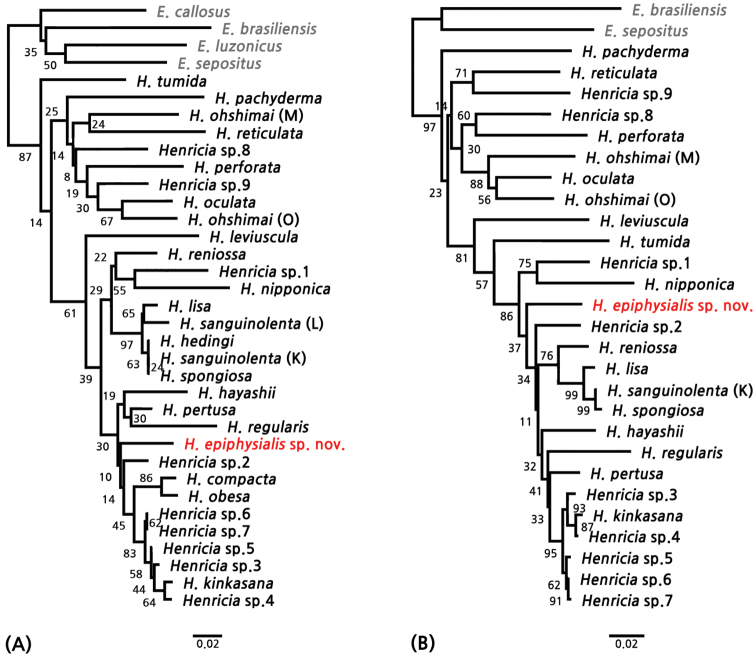
Phylogenetic trees of *Henricia* species including *Henricia epiphysialis* sp. nov. based on Neighbor joining (NJ) **A**NJ tree constructed with 189 bp of *COX1* dataset **B**NJ tree constructed with 537 bp of *COX1* dataset; K, Knott et al., 2018; L, Lopes et al., 2016; M, Misaki, Kanagawa in Wakita et al., 2019; O, Oshoro, Hokakido in Wakita et al., 2019.

We determined the phylogenetic relationships based on two *COX1* datasets with 27 and 31 species of the genus *Henricia* respectively, including *H. epiphysialis* sp. nov., on the basis of 189 and 537 bp of the mitochondrial *COX1* gene by using the NJ method. All *Henricia* species formed a monophyletic group with congeneric species and were clearly distinguished from the sister taxa (Fig. 5). *Henricia epiphysialis* sp. nov. was clearly monophyletic in the *COX1* dataset of 189 bp (Fig. 5A), but the 189 bp *COX1* dataset was too short for species-level identification using DNA barcoding analysis. Therefore, we reanalyzed phylogenetic relationships using a longer *COX1* dataset (537 bp) including 27 species of *Henricia* (Table 1, Fig. 5B). The phylogenetic tree of the 537 bp dataset also revealed that *H. epiphysialis* sp. nov. presented a monophyletic node according to short and long *COX1* datasets analysis (Fig. 5). In addition, each *Henricia* species examined was grouped at the species level. The intra- and inter-specific variations of *Henricia* species from the 537 bp *COX1* dataset were calculated by the Kimura 2-parameter model and presented in Table 3. According to the p-distance result, the average value between *Henricia* spp. and *Echinaster* spp. was 0.208 (Table 3). The range of inter-specific distance in the genus *Henricia* was 0.002–0.188, and the average value of inter-specific distance was 0.110 (Table 4). The inter-specific values of *H. kinkasana* and *Henricia* sp. 3–7 was lower than the relationships of other *Henricia* species (Table 3, 4). The range of *Henricia* species, except for *H. kinkasana* and *Henricia* sp. 3–7, was 0.040–0.188 and the average was 0.114 (Table 4). The range and average of *H. epiphysialis* sp. nov. and other *Henricia* species were 0.056–0.154 and 0.098, respectively (Table 4). Therefore, the molecular analysis results show that *H. epiphysialis* sp. nov. is a new species in the genus *Henricia*.

**Table 2.** Comparison of the morphological characteristics of *Henricia epiphysialis* sp. nov. with related *Henricia* species. Morphological data derived from the respective original descriptions, the present study, and Xiao et al. (2011).

	*Henricia epiphysialis* sp. nov.	*H. compacta* (Sladen, 1889)	*H. densispina* (Sladen, 1878)	*H. djakonovi* Chichvarkhin, 2017	*H. exigua* Hayashi, 1940	*H. kinkasana* Hayashi, 1940	*H. leviuscula* (Stimpson, 1857)	*H. regularis* Hayashi, 1940	*H. reniossa* Hayashi, 1940	*H. skorikovi* Djakonov, 1950
R/r	4.9–5.4	4.4–7.5	2.9–5.5	4.7–5.0	4.2–4.5	4.0–4.8	5.0–6.0	3.1–5.0	5.5–8.0	3.6–5.0
Number of spines of abactinal plates	11–40	up to 45	30 or more	20–30	5–13	5–18	40–60	9–20	15–60 or more	up to 16
Shape of abactinal spines	hooked crown	stout	granular	stout, barrel, bullet tip	slender, pointed tip	fine, slender, pointed tip	short, granular, solid glassy tip	slender, pointed tip	very fine, rough tip	short, robust, thorny tip
Number of abactinal papulae	1–3	1–3	1–3, rarely 5	1 or 2	1–3	single	1–3	single	1 or 2	2–6
Shape of abactinal plates	reniform with conspicuous epiphyseal ossicle	crescentic, compact	subtriangular	cross, oval, triangular, irregular	elliptic, quasi-triangular, quasi-quadrate	crescentic	elliptic, reniform, subquadrate	subquadrate	reniform	slender, rod-like
Shape of actinal plates	rounded cross, elongated cross	quadrilobed, squarish	elongated cross	square pillow	rounded cross, elongated cross	rounded cross, elongated cross	elongated cross, small rod-like	rounded cross	elongated cross, quasi-quadrate	elongated cross, rod-like
Number of actinal papulae	single	single	single	single	single	single	single	single	1 or 2	unknown
Number of adambulacral spines	10–14	5 or 6	11–16	10 or 11	13–15	8–12	15–18	9–13	15–25	7–12
Number of furrow spines	single	2 or 3	single	single	double	single	single	single or double	single	single
Pattern of adambulacral spines (near ambulacral furrow + near ventrolateral plate)	1 long, 2 slender, bluntly pointed + 4–14 slightly shorter	2 or 3 prominent + 4–6 slightly shorter	2 or 3 prominent, bluntly pointed + 4–16 slightly shorter	2 or 3 larger + 4–11 slightly shorter	1 long, 2 spatulate + 4–15 shorter	1 long, 2 slender, bluntly pointed + 4–12 slightly shorter	1 long, 3 stout + 5–18 slightly shorter	1 long, 2 slender, bluntly pointed + 4–13 slightly shorter	1 long, 5 slender + 7–25 slightly shorter	1 long, 2 coarse + 4–12 slightly shorter
Distribution	Korea (East Sea)	southern Australia	Bohai Sea, Yellow Sea, Korea Strait, Tatar Strait, Kurile Island, Japan, Philippines	Rudnaya Bay, Kievka Bay	southern Japan, East China Sea	Japan (off Kinkasan)	Korea (East Sea), Alaska (Kadiak)	East China Sea, Korea (East Sea, Korea Strait, Jeju Island), Japan (Goto Island, Uraga Channel)	Korea (East Sea), Japan (Yezo Strait)	White Sea, Barents Sea, Chesha Bay

**Table 3.** Pairwise genetic comparison for 537 bp of the mitochondrial *COX1* gene in 27 species of *Henricia* including *Henricia epiphysialis* sp. nov.

Species	1	2	3	4	5	6	7	8	9	10	11	12	13	14	15	16	17	18	19	20	21	22	23	24	25	26	27	28
*H. epiphysialis* sp. nov.																												
*H. hayashii*	0.069																											
*H. kinkasana*	0.065	0.056																										
*H. leviuscula*	0.141	0.143	0.132																									
*H. lisa*	0.084	0.069	0.054	0.114																								
*H. nipponica*	0.096	0.114	0.112	0.157	0.096																							
*H. oculata*	0.131	0.143	0.134	0.150	0.139	0.161																						
*H. ohshimai* 1	0.129	0.143	0.141	0.149	0.136	0.150	0.064																					
*H. ohshimai* 2	0.142	0.166	0.151	0.161	0.159	0.170	0.079	0.094																				
*H. pachyderma*	0.154	0.161	0.159	0.160	0.154	0.188	0.136	0.145	0.133																			
*H. perforata*	0.152	0.164	0.152	0.154	0.167	0.165	0.117	0.141	0.138	0.143																		
*H. pertusa*	0.067	0.048	0.036	0.136	0.061	0.105	0.141	0.138	0.151	0.173	0.166																	
*H. regularis*	0.090	0.083	0.071	0.145	0.085	0.118	0.152	0.138	0.165	0.188	0.191	0.065																
*H. reniossa*	0.077	0.058	0.056	0.114	0.042	0.096	0.136	0.134	0.159	0.154	0.169	0.052	0.083															
*H. reticulata*	0.135	0.133	0.133	0.171	0.152	0.173	0.120	0.122	0.113	0.131	0.159	0.133	0.146	0.147														
*H. sanguinolenta*	0.084	0.067	0.056	0.118	0.013	0.092	0.139	0.134	0.159	0.154	0.164	0.063	0.090	0.034	0.150													
*H. spongiosa*	0.088	0.071	0.061	0.118	0.017	0.092	0.144	0.139	0.164	0.159	0.169	0.067	0.094	0.038	0.154	0.004												
*H. tumida*	0.104	0.117	0.115	0.161	0.124	0.135	0.137	0.137	0.146	0.156	0.165	0.120	0.133	0.124	0.128	0.124	0.129											
*Henricia* sp. 1	0.090	0.087	0.077	0.141	0.071	0.086	0.150	0.145	0.162	0.150	0.156	0.081	0.111	0.058	0.160	0.067	0.071	0.133										
*Henricia* sp. 2	0.062	0.062	0.044	0.120	0.059	0.105	0.141	0.145	0.158	0.159	0.154	0.050	0.081	0.056	0.147	0.056	0.061	0.113	0.077									
*Henricia* sp. 3	0.058	0.052	0.015	0.123	0.052	0.105	0.136	0.136	0.156	0.156	0.159	0.032	0.069	0.048	0.140	0.055	0.059	0.122	0.073	0.040								
*Henricia* sp. 4	0.063	0.054	0.006	0.129	0.052	0.110	0.131	0.138	0.149	0.156	0.154	0.034	0.069	0.054	0.137	0.055	0.059	0.113	0.075	0.042	0.009							
*Henricia* sp. 5	0.058	0.048	0.015	0.127	0.050	0.103	0.141	0.138	0.154	0.152	0.164	0.029	0.058	0.048	0.138	0.053	0.057	0.120	0.073	0.044	0.011	0.013						
*Henricia* sp. 6	0.056	0.048	0.015	0.123	0.046	0.099	0.136	0.134	0.149	0.149	0.164	0.029	0.058	0.044	0.140	0.048	0.053	0.117	0.069	0.040	0.011	0.013	0.004					
*Henricia* sp. 7	0.058	0.050	0.017	0.125	0.048	0.096	0.134	0.131	0.147	0.147	0.161	0.031	0.061	0.046	0.138	0.050	0.055	0.120	0.067	0.042	0.013	0.015	0.006	0.002				
*Henricia* sp. 8	0.141	0.145	0.136	0.131	0.141	0.147	0.096	0.107	0.111	0.145	0.107	0.138	0.147	0.138	0.143	0.134	0.138	0.147	0.138	0.141	0.141	0.134	0.136	0.132	0.129			
*Henricia* sp. 9	0.145	0.143	0.145	0.148	0.157	0.176	0.107	0.127	0.139	0.147	0.129	0.154	0.166	0.150	0.105	0.152	0.157	0.146	0.168	0.145	0.138	0.138	0.148	0.148	0.150	0.125		
*E. brasiliensis*	0.220	0.212	0.228	0.210	0.217	0.207	0.227	0.217	0.202	0.190	0.200	0.230	0.230	0.233	0.202	0.220	0.225	0.197	0.217	0.227	0.225	0.230	0.225	0.223	0.220	0.205	0.217	
*E. sepositus*	0.192	0.190	0.207	0.190	0.207	0.190	0.192	0.195	0.183	0.214	0.192	0.207	0.215	0.210	0.219	0.205	0.205	0.204	0.209	0.190	0.210	0.205	0.210	0.205	0.205	0.178	0.212	0.190

**Table 4.** The range and average p-distance values of *Henricia* species examined in this study.

Group	Range	Average
*H. epiphysialis*–other *Henricia* sp.	0.056–0.154	0.098
All of *Henricia* species	0.002–0.188	0.110
All *Henricia* species except for *H. kinkasana* and *Henricia* sp.3–sp.7	0.040–0.188	0.114

## Discussion

The diagnostic combination of the morphological characteristics of the genus *Henricia* such as spination of the abactinal (primarily on the disk and proximal portion of the arm), adambulacral, and oral plates was found to be consistent and reliable for determining the species (e.g., Hayashi 1940; Djakonov 1950; Clark and Jewett 2010). The shape of the abactinal spines and plate formation exhibited the distinct morphological characteristics of *Henricia epiphysialis* sp. nov. (Figs 3A, C, D, 4A–C). *Henricia epiphysialis* sp. nov. was compared with nine related *Henricia* species (Table 2). This new species has very peculiar abactinal spines that form a hooked crown and can be compared with the robust, coarse abactinal spines of *H. compacta*, *H. leviuscula*, *H. skorikovi*. *Henricia epiphysialis* sp. nov. is superficially similar to *H. leviuscula* in having short and coarse abactinal spines, but differs mainly in the arrangement of the abactinal paxillae (*H. epiphysialis* sp. nov. has less dense abactinal paxillae, whereas *H. leviuscula* has dense abactinal paxillae) and formation of abactinal spines (*H. epiphysialis* sp. nov. has spines forming a hooked crown with small connected apical tips, whereas *H. leviuscula* has spines with solid glassy tips). *Henricia epiphysialis* sp. nov. is morphologically distinguishable from its congeners primarily by the presence of conspicuous epiphyseal ossicles in almost every plate, and also by the distinctive arrangement of the epiphyseal ossicles of the ventrolateral plates, forming a knob-like connection to the adambulacral plates (Fig. 3D). The knob-like form of epiphyseal ossicles in the ventrolateral plates is rarely seen in related *Henricia* species having slender arms with imbricated plates. Our new species, *H. epiphysialis* sp. nov. is morphologically closer to *H. reniossa*: they share similar reniform abactinal plates, elongated cross shaped actinal plates, but *H. epiphysialis* sp. nov. possessed well-developed epiphyseal ossicles in both abactinal and actinal plates. The molecular analysis supports the morphological similarity by showing both species in the same clade (Fig. 5B).

Other morphological characteristics of *H. epiphysialis* sp. nov., such as the ratio of arm to disk and number of adambulacral spines, are similar to those of *H. kinkasana* which is a slender-rayed species; however, this new species differs mainly in the number of abactinal spines and shape of both abactinal and lateral spines. *Henricia epiphysialis* sp. nov. has 11–40 robust abactinal spines on the abactinal plate, whereas *H. kinkasana* has five to 18 fine, delicate abactinal spines. Moreover, the conspicuous epiphyseal ossicles at the ends of the abactinal and lateral plates are exclusively present in *H. epiphysialis* sp. nov. Therefore, the extension of ossicles in the plate and hooked crown shape of the spines are diagnostic characteristics for this new species.

In this study, we identify a new *Henricia* species based on its morphological characteristics and DNA barcoding. *Henricia epiphysialis* sp. nov. has distinct morphological features and was classified as a new species after comparison with related species. Moreover, the molecular analysis showed that *H. epiphysialis* sp. nov. clearly formed a monophyletic node in a large clade of the genus *Henricia* species (Fig. 5), and the minimum value for the inter-specific distance was significantly higher than the inter-specific distance reported in a previous asteroid DNA barcoding study (Table 3) (Ward et al. 2008). Therefore, the molecular analysis clearly supported the diagnostic morphological identification of *H. epiphysialis* sp. nov. as a new species under the genus *Henricia*. The mitochondrial *COX1* gene is especially useful and effective for the DNA barcoding analyses of *Henricia* species.

## Supplementary Material

XML Treatment for Henricia epiphysialis
